# Injury and local injection and the risk of foot/ankle osteoarthritis: a case–control study in retired UK male professional footballers

**DOI:** 10.1093/rheumatology/keaf518

**Published:** 2025-10-22

**Authors:** Ahmed Ali Thanoon, Shima Espahbodi, Monirah Ali Shuaib, Bonnie Millar, Ashley Duncan, Catherine J Bowen, Terence W O’Neill, Richard J Wakefield, Fiona E Watt, David A Walsh, Gordon Fuller, Mark E Batt, Sanjay M Parekh, Gwen Sascha Fernandes, Michael Doherty, Weiya Zhang

**Affiliations:** Academic Rheumatology, Injury Inflammation and Recovery Sciences, School of Medicine, University of Nottingham, Nottingham, UK; Versus Arthritis Centre for Sport, Exercise and Osteoarthritis, University of Nottingham, Nottingham, UK; Pain Centre Versus Arthritis, University of Nottingham, Nottingham, UK; Academic Rheumatology, Injury Inflammation and Recovery Sciences, School of Medicine, University of Nottingham, Nottingham, UK; Versus Arthritis Centre for Sport, Exercise and Osteoarthritis, University of Nottingham, Nottingham, UK; Pain Centre Versus Arthritis, University of Nottingham, Nottingham, UK; Academic Rheumatology, Injury Inflammation and Recovery Sciences, School of Medicine, University of Nottingham, Nottingham, UK; Versus Arthritis Centre for Sport, Exercise and Osteoarthritis, University of Nottingham, Nottingham, UK; Pain Centre Versus Arthritis, University of Nottingham, Nottingham, UK; Academic Rheumatology, Injury Inflammation and Recovery Sciences, School of Medicine, University of Nottingham, Nottingham, UK; Pain Centre Versus Arthritis, University of Nottingham, Nottingham, UK; NIHR Nottingham Biomedical Research Centre, Nottingham University Hospitals, NHS Trust, University of Nottingham, Nottingham, UK; National Institute for Health Research ARC EM, University of Nottingham, Nottingham, UK; Health Sciences, Faculty of Environmental and Life Sciences, University of Southampton, Southampton, UK; NIHR Manchester Biomedical Research Centre, Manchester Academic Health Sciences Centre, Manchester University Foundation NHS Trust, Manchester, UK; NIHR Leeds Biomedical Research Centre, University of Leeds, Leeds, UK; Department of Immunology and Inflammation, Imperial College London, London, UK; Academic Rheumatology, Injury Inflammation and Recovery Sciences, School of Medicine, University of Nottingham, Nottingham, UK; Pain Centre Versus Arthritis, University of Nottingham, Nottingham, UK; NIHR Nottingham Biomedical Research Centre, Nottingham University Hospitals, NHS Trust, University of Nottingham, Nottingham, UK; Advanced Pain Discovery Platform, Academic Rheumatology, University of Nottingham, Nottingham, UK; Centre for Urgent and Emergency Research, University of Sheffield, Sheffield, UK; Versus Arthritis Centre for Sport, Exercise and Osteoarthritis, University of Nottingham, Nottingham, UK; Perspectum, Oxford, UK; Academic Rheumatology, Injury Inflammation and Recovery Sciences, School of Medicine, University of Nottingham, Nottingham, UK; South West Critical Thinking Unit, Population Health Directorate, NHS England, Bristol, UK; Academic Rheumatology, Injury Inflammation and Recovery Sciences, School of Medicine, University of Nottingham, Nottingham, UK; Versus Arthritis Centre for Sport, Exercise and Osteoarthritis, University of Nottingham, Nottingham, UK; Pain Centre Versus Arthritis, University of Nottingham, Nottingham, UK; Academic Rheumatology, Injury Inflammation and Recovery Sciences, School of Medicine, University of Nottingham, Nottingham, UK; Versus Arthritis Centre for Sport, Exercise and Osteoarthritis, University of Nottingham, Nottingham, UK; Pain Centre Versus Arthritis, University of Nottingham, Nottingham, UK; NIHR Nottingham Biomedical Research Centre, Nottingham University Hospitals, NHS Trust, University of Nottingham, Nottingham, UK

**Keywords:** foot/ankle OA, injury, injection, professional footballers

## Abstract

**Objective:**

The objective of this study was to examine whether foot/ankle injury and injection contribute to the risk of foot/ankle OA in retired UK male professional footballers.

**Methods:**

This was a case–control study among retired UK male footballers, in which cases reported General Practitioner–diagnosed foot/ankle OA or forefoot/ankle surgery after retirement, and controls reported neither. Injury was defined as significant foot/ankle injury with pain for most days over 3 months during their career. Injection was defined as injection of corticosteroids or other agents into foot/ankle joints during their career. Adjusted odds ratios (aORs) with 95% confidence interval (CIs) were calculated using logistic regression. Areas Under the Curve (AUCs) and 95% CIs were estimated to examine the contribution of injury and/or injection in the context of other available risk factors.

**Results:**

Of 424 footballers studied, 63 had foot/ankle OA and 361 had neither. Cases had similar mean age (63.2 *vs* 63.0, *P = *0.457) and BMI (27.7 *vs* 27.0, *P = *0.240) to those of controls, but more foot/ankle injury (73.3% *vs* 42.5%, *P < *0.001) and injections (75.0% *vs* 48.4%, *P < *0.001), with aORs of 4.23 (95% CI 1.88–9.48) and 2.62 (95% CI 1.19–5.78), respectively. The AUC was 0.69 (95% CI 0.62–0.77) for injury, 0.74 (95% CI 0.66–0.81) for injury and injection, and 0.78 (95% CI 0.70–0.85) for all risk factors. Similar results were observed in footballers with ankle OA only.

**Conclusion:**

Injury was a major risk factor for foot/ankle OA in retired UK male professional footballers. The role of injection needs cautious interpretation due to potential confounding by indication.

Rheumatology key messages

- Foot/ankle injury is a major risk factor that is independently associated with foot/ankle OA in retired male professional footballers.
- Professional footballers should consider preventive strategies for football-related injury to minimize the likelihood of developing foot/ankle OA.
- Further research is needed to test the association between corticosteroid joint injections and the risk of developing foot/ankle OA in professional footballers.

## Introduction

Professional football is a high-speed contact sport that carries a high risk of injury [1, 2]. Foot/ankle injuries are particularly common, with ankle sprains being the most frequent ankle injury and metatarsal fractures the most common foot injury [3]. These injuries occur more often during football matches than during training sessions, due to the high speed, jumping, frequent changes in direction, and competitive nature of the game [1]. Joint injury can lead to pain, swelling and damage to the articular cartilage and other joint tissues [4] and result in subsequent foot/ankle OA with chronic pain, disability and reduced quality of life [1, 5, 6].

Although injury is highly prevalent in male professional footballers, the widespread use of injection therapies such as corticosteroids, local anaesthetics, platelet-rich plasma, and hyaluronic acid remains controversial, due to concerns about their long-term effects and limited evidence supporting their efficacy [7, 8]. Injections can alleviate pain and enable professional footballers to return to play more quickly [9]. However, they may mask underlying joint damage, and therefore potentially accelerate joint structural deterioration over time [9]. The frequent use of injections raises concerns about exacerbating cartilage damage, particularly when combined with the high physical demands required in professional football [7].

We have recently shown that retired male professional footballers are at higher risk of having foot/ankle OA than male controls in the general population [10]. However, specific risk factors for foot/ankle OA (e.g. potentially foot/ankle injury, frequently associated with foot/ankle injection) and their contribution to the development of foot/ankle OA in the later life of professional footballers remain poorly understood.

To address this gap, we undertook the current study, which aimed to examine whether foot/ankle injury and injection independently contribute to the risk of foot/ankle OA in male retired professional footballers and to determine the total risk contribution of foot/ankle injury and injection in the context of other recognized risk factors for OA.

## Methods

### Study design

This was a case–control study within the Foot/ankle Osteoarthritis and Cognitive impairment in retired UK Soccer players (FOCUS) study conducted in the UK between August 2020 and October 2021 and followed its published protocol [11]. The FOCUS study measured both foot/ankle OA and neurodegenerative outcomes in retired male professional footballers and in male controls in the general population, and the neurodegenerative findings have been published separately [12, 13]. The findings for the prevalence of foot/ankle OA in footballers and controls will also be reported separately but currently are available in abstract form [10].

### Participants

**Figure 1.**
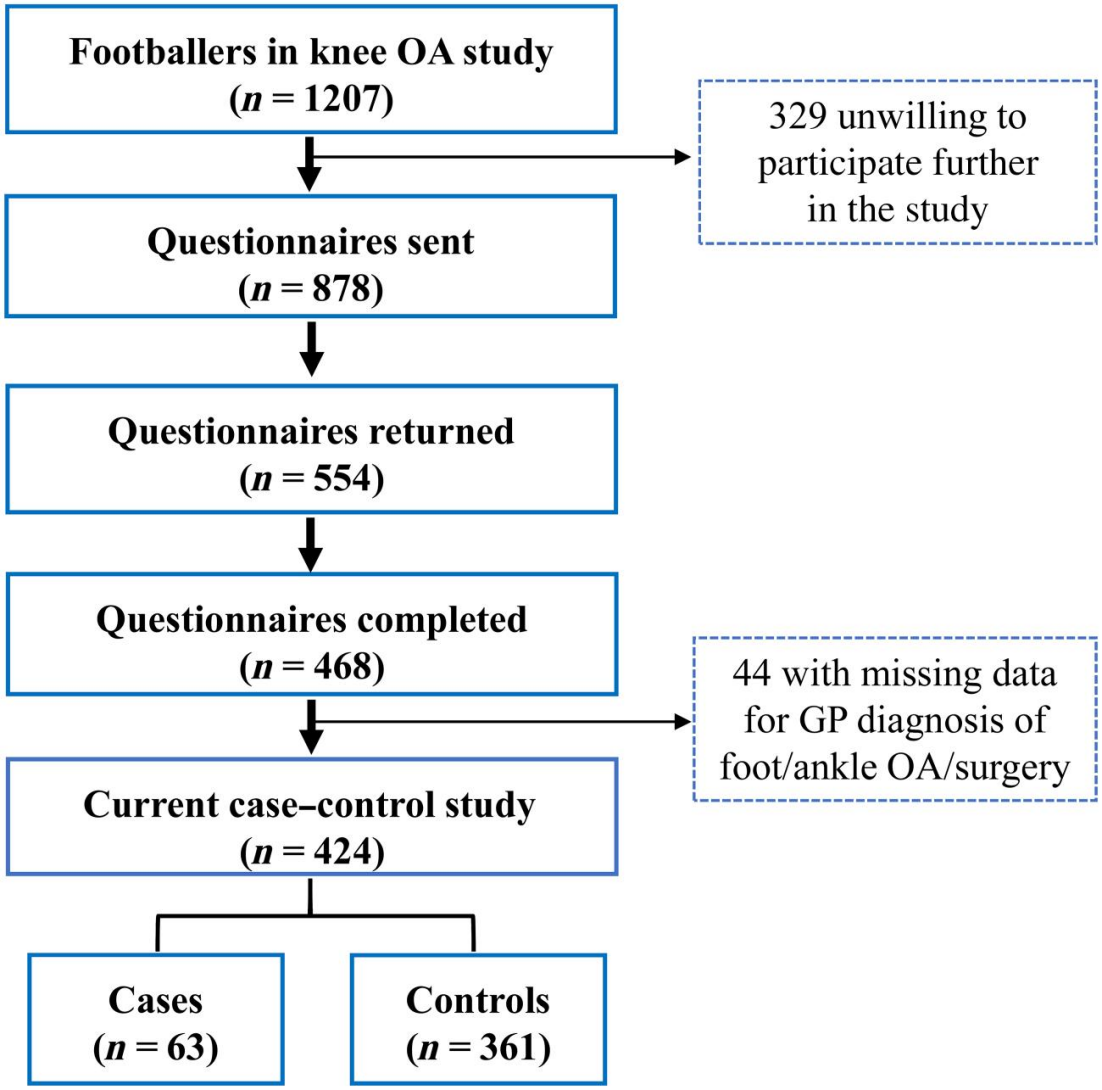
Recruitment of retired male professional footballers. Of 1207 footballers in the previous knee OA study, 878 were sent questionnaires. Of 554 returned questionnaires, 468 were completed, 44 were excluded due to missing data on general practitioner–diagnosed foot/ankle OA or forefoot/ankle surgery, leaving 424 eligible participants. Of these, 63 were classified as cases and 361 as controls

Participants in the FOCUS study were recruited from our previous study examining the prevalence of knee OA (KOA) in retired male professional footballers living throughout the UK [14]. Of the 1207 retired professional footballers who participated in the KOA study, 878 professional footballers indicated their willingness to participate in future research and were the source populations for the FOCUS study (Fig. 1).

Inclusion criteria for professional footballers were males aged 40 years and over living in the UK who had played professionally in the top four tiers (Premier League, Championship, League One, League Two) of the English Football League. All professional footballers could read and write in English. Exclusion criteria included having a terminal illness or being unable to provide written informed consent or complete a questionnaire.

### Exposures

Foot/ankle injury was the primary exposure assessed via a self-completed postal questionnaire. Participants were first asked: ‘Have you ever had a significant football-related injury to your ankles, feet, toe(s)?’ A significant injury was defined as ‘one which caused you pain for most days for at least a 3-month period and resulted in an absence from all training and matches during this time’. Response options were (Yes/No). Those who answered ‘Yes’ were then asked to indicate the total number of significant football-related injuries they had sustained throughout their professional career to specific regions of the foot and ankle (right/left ankle, mid-foot, big toe, and other toes). Since we could not distinguish between joint and peri-articular soft-tissue injuries, foot/ankle injury was used as a generic term.

In relation to foot/ankle injury, foot/ankle injection was also assessed during their playing career using the two questions, ‘Have you ever had any injections into your ankles?’ and ‘Have you ever had injections anywhere else in your foot other than in your ankle(s)? If yes, please state where and how many’. If they replied positively to having injections in their ankles, they were then asked to identify which ankle was injected and the type of injection used, with the following options: corticosteroids (cortisone); anaesthetics (lignocaine); do not know; or other (please specify). Since we did not distinguish between intra-articular or extra-articular injections, foot/ankle injection was used as a generic term. Footballers were also asked to report the greatest number of corticosteroid  injections they had received into each ankle during any one season. For this analysis, only participants who indicated receiving corticosteroid injections were included, ensuring the injection variable reflected corticosteroid exposure specifically.

### Radiographic assessment of foot/ankle OA

Footballers who returned completed questionnaires, indicated willingness to undergo foot/ankle radiographic assessment and lived reasonably close to one of five collaborating NHS hospitals (Nottingham, Manchester, Leeds, Southampton and London) were invited to attend a single hospital radiology appointment for bilateral foot and ankle radiographs, irrespective of self-reported foot/ankle OA outcomes [11]. All five hospitals used the same radiographic protocol [15]. For the dorsoplantar foot view, participants stood on the detector with their feet flat and the X-ray tube angled 15° upward, with a vertical central ray centred on the third metatarsal base. For the lateral view, participants stood on a low platform with the detector positioned on the side of their foot with the X-ray tube angled at 90° with a central horizontal ray centred on the first metatarsal [15]. For the anteroposterior ankle view, the ankle was dorsiflexed 90° with the leg rotated 20° medially until medial and lateral malleoli were equidistant from the image receptor. Each ankle was assessed separately [15].

Using the La Trobe radiographic atlas, foot radiographic OA (ROA) was defined primarily as definite osteophyte (OST ≥2) or joint space narrowing (JSN ≥2) in any of the five foot joints included in the atlas, specifically, in the forefoot the first MTP joint, and in the midfoot the first cuneo-metatarsal joint (CMJ), second CMJ, navicular-first cuneiform joint (N1stCJ), and talo-navicular joint (TNJ), in either dorsoplantar or lateral views [11, 15]. Ankle ROA was defined by the presence of either OST ≥2 or JSN ≥2 in at least one ankle (talocrural) joint in either the anterior–posterior or lateral view [15]. Symptomatic radiographic OA (sROA) was defined as presence of ROA in a joint and pain in the same region as the joint for most days in the past month, using the questionnaire line-drawing foot manikin [16].

All radiographic images were read by a single observer (A.T.), using RadiAnt DICOM Viewer software 2021 [17]. To establish intra-rater and inter-rater reliability for scoring OA, radiographs from 60 randomly selected participants were re-scored 2 weeks later by A.T. and were also scored by a second assessor (M.S.) blinded to the original scores. The procedure was repeated three times with 20 different participants at each time point (starting, midpoint, and end of the study), resulting in evaluation of 120 feet and ankles in 60 participants [18].

### Outcome measures

The primary outcome was self-reported foot/ankle OA, defined as either foot/ankle OA diagnosed by a doctor/general practitioner (GP), or a history of forefoot/ankle surgery performed at age 40 years or above. The diagnosis was based on responses to a closed question about GP-diagnosed OA, and information on surgery was obtained from questions on anatomical site (ankle or forefoot) and age, with only surgery reported at age ≥40 years being included. The age threshold of 40 was selected based on the average of retirement ages to avoid surgery undertaken for acute injury (largely peri-articular) during their career period, rather than because of OA [19]. Footballers with self-reported foot/ankle OA formed the case group of this study and the remaining participants who did not fulfil this definition formed the control group.

The secondary outcome was sROA foot/ankle, defined as those with both ROA and pain in the same region as the joint with ROA, involving any of the assessed joints in the foot/ankle. Footballers with sROA foot/ankle formed the case group for this study and the remaining participants formed the control group.

### Covariates

Covariates included demographic, constitutional, and biomechanical risk factors, as well as career details of the footballers [11]. These included age, body mass index (BMI), current hallux valgus (determined using a validated line drawing) [20], a pattern 3 index-to-ring finger length (2D:4D) ratio [21] and nodal OA status, again determined using validated diagrams [22]. Nodal OA, a strong risk factor for generalized OA, was classified as present in those reporting nodes on at least two rays of both hands [22]. Gout, reported as a self-reported physician diagnosis, was included as a covariate, due to its reported association with OA of the foot, especially the first metatarsophalangeal joint (MTPJ) and mid-foot [23]. Flat-footedness was defined as present if a subject had been informed of this by a health professional. It was considered a potential risk factor for midfoot OA, as part of the foot/ankle joint complex, due to altered biomechanics and increased joint loading that might lead to OA [24]. Comorbidity was assessed using the Charlson Comorbidity Index [25], and deprivation was assessed by the Index of Multiple Deprivation (categorized as deciles) and derived from the participant’s postcode [26]. Further details of the questionnaire are available in the published protocol [11].

Footballers were also asked about their career training dose, career duration, weekly training hours, total matches played, and positions played (such as goalkeeper, defender, midfielder and forward) [11]. Career duration was calculated from the time between the start of a professional football career (i.e. signed up with a professional football club) and the date of retirement from playing professional football (end of contract or retirement). Career training was estimated by multiplying the number of hours played per week by 40 weeks (the average season) and by career duration. Weekly training hours were determined by multiplying the number of hours trained per day by the number of training days per week [11].

### Statistical analysis

The sample size was calculated based on a 57.5% frequency of ankle injury in the retired male professional footballers [27] and a minimum clinically important OR of 3 for foot/ankle OA after foot/ankle injury [2, 28]. As a result, a minimum of 61 participants per group was required for this study to achieve 90% power with 5% Type I error (i.e. a significance level of 0.05). The calculation was performed using a *z*-test and a multiple logistic regression model with an *R*^2^ of 0.3 for multiple covariates, as implemented in G*Power V.3.1.9.2 [29].

Descriptive analyses were conducted using the χ^2^ test for categorical variables, the independent *t* test for normally distributed continuous variables, and the Mann–Whitney *U* test for non-normally distributed continuous variables. Imputation was not performed, because the overall proportion of missing data across variables was <10% [30].

Logistic regression was used to calculate the adjusted odds ratio (aOR) and 95% confidence interval (CI). A multivariable logistic regression analysis was conducted using a backward stepwise approach, where all available risk factors, irrespective of their significance, were initially included in the model. This approach ensured that all putative risk factors were considered at the first step. The variables included were injury, injection, age, BMI, socio-economic status, Charlson Comorbidity Index, nodal OA, gout, pattern 3 2D:4D ratio, current hallux valgus, flat feet, career training dose, career duration, total matches played, position played, and training hours per week. Non-significant variables (*P* > 0.05) were subsequently removed from the model one at a time, starting with the least significant, until all variables within the model remained statistically significant. The remaining variables were confirmed as independent risk factors for the outcome measure [31].

The Area Under the Curve (AUC) and 95% CI were calculated using the Receiver Operating Characteristic (ROC) analysis [32]. The AUC reflects the discriminative power of risk factors included in the logistic regression model, with values ranging from 0 to 1. The higher the AUC, the better the discriminative power of the model to separate cases and controls. We started with injury alone, then added injection and other risk factors gradually into the model to see the change in AUC. In addition to injury and injection, all covariates included in the logistic regression were also included into the model to calculate the AUC of the full model. This would allow us to determine the relative contribution of injury and injection in the context of all available risk factors.

The main analysis was undertaken for self-reported foot/ankle OA. A subgroup analysis was also conducted for self-reported ankle OA alone. A sensitivity analysis was performed for GP-diagnosed foot/ankle OA only, excluding surgery at age ≥40 years. Another sensitivity analysis was performed for foot/ankle sROA.

All analyses were conducted using the Statistical Package for the Social Sciences (SPSS), version 29. The *P*-value of 0.05 was used as a threshold for statistical significance.

## Results

Of 878 footballers who had previously indicated willingness to be approached for future research, 468 (53%) completed and returned the postal questionnaire. Of these, 424 (48%) were eligible for inclusion in the case–control analysis, comprising 63 cases and 361 controls. Among the cases, 45 reported GP-diagnosed foot/ankle OA and 18 reported forefoot/ankle surgery at age ≥40 years (Fig. 1). There were no significant differences in age, BMI, or socio-economic status between questionnaire responders and non-responders (Supplementary Table S1).

Cases played more matches during their careers compared with controls (532.0 *vs* 454.8, respectively; *P = *0.028). In addition, a greater proportion of cases than controls had experienced foot/ankle injury (73.3% *vs* 42.5%, respectively; *P < *0.001) and had reported a history of any type of foot/ankle injection (75.0% *vs* 48.4%, respectively; *P < *0.001), any ankle injection (61.2% *vs* 36.6%, respectively; *P < *0.001), and ankle corticosteroid injection (57.1% *vs* 32.1%, respectively; *P < *0.001). The maximum number of ankle corticosteroid injections per season was 22 in cases and 12 in controls, with means of 4.2 and 3.11, respectively. Furthermore, cases were also more likely to have nodal OA (16.1% *vs* 5.3%; *P = *0.002), gout (19.0% *vs* 10.2%; *P = *0.044) and current hallux valgus (36.0% *vs* 22.7%; *P = *0.026) compared with controls. No other differences were observed between cases and controls (Table 1).

**Table 1.** Characteristics of footballers with (cases) and without (controls) foot/ankle OA

	Cases (*n* = 63)	Controls (*n* = 361)	*P*-value
Age (years), mean (s.d.)	63.22 (9.25)	63.08 (10.57)	0.457^g^
BMI (kg/m^2^), mean (s.d.)	27.70 (3.85)	27.09 (2.97)	0.240^g^
Socio-economic decile, median (IQR)	8.00 (6-9)	8.00 (6-10)	0.711^h^
Charlson Comorbidity Index, mean (s.d.)	1.25 (2.03)	1.06 (2.14)	0.498^g^
Nodal OA, *n*/total (%)	10/62 (16.13)	19/356 (5.34)	**0.002** ^i^
Gout, *n*/total (%)	12/63 (19.05)	37/361 (10.25)	**0.044** ^i^
Pattern 3 2D:4D finger ratio, *n*/total (%)	43/62 (69.35)	228/350 (65.14)	0.519^i^
Current hallux valgus, *n*/total (%)	22/61 (36.07)	81/356 (22.75)	**0.026** ^i^
Flat feet, *n*/total (%)	7/61 (11.48)	29/342 (8.48)	0.450^i^
Foot/ankle injury, *n*/total (%)	44/60 (73.33)	140/329 (42.55)	**<0.001** ^i^
Foot/ankle injection (any injectable)^a^, *n*/total (%)	45/60 (75.00)	155/320 (48.44)	**<0.001** ^i^
Foot injection, *n*/total (%)	18/58 (31.03)	62/324 (19.14)	**0.040** ^i^
Ankle injection, *n*/total (%)	38/62 (61.29)	126/344 (36.63)	**<0.001** ^i^
Ankle corticosteroids injections, *n*/total (%)	36/63 (57.14)	116/361 (32.13)	**<0.001** ^i^
Number of ankle corticosteroids injections per season, mean (maximum)	4.21 (22.00)	3.09 (12.00)	0.159^g^
Career training dose (hours) mean (s.d.)^b^	8407.00 (4444.81)	7608.34 (4020.70)	0.197^g^
Career duration (years), mean (s.d.)^c^	14.11 (4.48)	13.64 (5.57)	0.466^g^
Total matches played, mean (s.d.)^d^	532.03 (252.02)	454.89 (240.11)	**0.028** ^g^
Training hours/week, mean (s.d.)^e^	15.24 (6.18)	14.38 (5.03)	0.300^g^
Position played, *n* (%)			
Goalkeeper	9 (14.29)	321 (8.86)	
Outfielders^f^	54 (85.71)	329 (91.14)	0.179^i^
Defender	21 (33.33)	140 (38.78)	0.411^i^
Midfielder	17 (26.98)	85 (23.55)	0.556^i^
Forward	15 (23.81)	103 (28.53)	0.440^i^

a Includes any of the following: Corticosteroids or anaesthetics or ‘don’t know’.

b Career training dose calculated by multiplying hours per week trained, number of weeks per season (40), and career duration.

c Professional career duration is the difference in years between commencing and retiring from professional football.

d Number of matches played during the professional career.

e Hours per week calculated by multiplying hours per day and days per week.

f Outfielder was any player other than a goalkeeper.

g Independent sample *t* test.

h Mann–Whitney *U* test.

i χ^2^ test.

* *P* ≤ 0.05 is defined as significant and marked in bold. IQR: interquartile range.

Of the 16 available risk factors included in the logistic regression model, only two factors (injury and injection) remained significant (*P < *0.05) in the model after the backwards stepwise selection. The aORs (95% CI) were 4.23 (1.88–9.48) for foot/ankle injury and 2.62 (1.19–5.78) for foot/ankle injection, respectively (Table 2). Although there was a strong correlation between foot/ankle injury and injection (*P < *0.001), both remained in the model as statistically significant risk factors for foot/ankle OA.

**Table 2.** Multivariable logistic regression model for foot/ankle OA in footballers (backwards stepwise selection model)

	Adjusted OR (95% CI)	*P*-value
Foot/ankle injury	4.23 (1.88–9.48)	<0.001^a^
Foot/ankle injection^a^	2.62 (1.19–5.78)	0.017^a^

a Results derived from the last step when this variable remained in the model. OR: odds ratio.

The ROC analysis demonstrated a good discriminative power of injury on its own in the model (AUC 0.69, 95% CI 0.61–0.77). The AUC increased somewhat by adding injection into the model (AUC 0.73, 95% CI 0.66–0.80). The difference between the AUC with injury and injection and the AUC with all 16 available risk factors (AUC 0.77, 95% CI 0.70–0.85) was not significant, as demonstrated by the overlapping 95% CIs (Table 3).

**Table 3.** Area under the curve (AUC) for various risk factors included in the logistic regression model for foot/ankle OA

Risk factors included in the logistic regression model	AUC	95% CI
Injury	0.693	(0.616–0.771)
Injury and injection	0.736	(0.663–0.809)
Full model^a^	0.776	(0.703–0.850)

a The full model included all risk factors collected in this study, including injury, injection, age, BMI, socio-economic status, Charlson Comorbidity Index, nodal OA, gout, pattern 3 2D:4D digit ratio, current hallux valgus, flat feet, career training dose, career duration, total matches played, position played, and training hours per week.

Both intra-rater (weighted kappa ranged from 0.61 to 0.96) and inter-rater agreement (weighted kappa ranged from 0.83 to 0.95) were good to excellent for OST and JSN across all assessed joints. The sensitivity analysis was undertaken in 113 footballers who underwent radiographic assessment, in which footballers with foot/ankle sROA (*n* = 45) were compared with footballers without sROA (*n* = 68). The results showed that the aORs were 3.33 (95% CI: 1.33–8.45) for foot/ankle injury and 2.57 (95% CI: 0.93–8.11) for foot/ankle injection (Supplementary Table S2). The AUC values for injury and injection in those with sROA are presented in Supplementary Table S3. Subgroup analysis for ankle OA alone showed aORs (95% CI) of 2.77 (1.28–5.97) for ankle injury and 4.33 (2.02–9.29) for ankle CS injection, respectively (Supplementary Table S4). Similarly, the AUC values for injury and injection in those with ankle OA alone were comparable with those observed in foot/ankle OA (Supplementary Table S5). Similar results were observed in the sensitivity analysis using GP-diagnosed foot/ankle OA alone (excluding surgery), in which the aORs were 3.35 (95% CI: 1.47–7.63) for foot/ankle injury and 2.57 (95% CI 1.12–5.89) for foot/ankle injection, which were similar to the main results (Supplementary Table S6).

## Discussion

This study found that self-reported foot/ankle injury was significantly associated with the risk of foot/ankle OA in retired male professional footballers. Although foot/ankle injection of any kind was also associated with foot/ankle OA, this may reflect confounding by indication (i.e. injections were indicated by injury). The study also found that 74% of cases and controls can be successfully separated by just two major risk factors, namely, foot/ankle injury and foot/ankle injections. This separation power was close to the 78% obtained using all available risk factors for OA, suggesting: (1) the majority of the risk of foot/ankle OA in professional footballers may be attributed to foot/ankle injury and subsequent injection; (2) injection is likely to be a surrogate marker of injury rather than an independent risk factor.

Injury is a well-established risk factor for OA across various populations, with higher risks observed in male professional footballers compared with male controls from the general population [2, 28, 33, 34]. Among UK professional footballers, joint injury has been associated with significantly higher odds of knee OA (aOR 3.51, 95% CI 1.94–6.32) [2] and hip OA (aOR 10.2, 95% CI 2.1–48.8) [33]. Similarly, retired Olympians from multiple countries, primarily those competing in high-impact sports (e.g. athletics, weightlifting, and combat sports) have been reported to have an increased risk of knee OA (aOR 9.40, 95% CI 6.90–12.79), hip OA (aOR 14.30, 95% CI 8.25–24.79) and ankle OA (aOR 9.90, 95% CI 5.05–19.41) [34]. In the United States Johnston County Osteoarthritis Study, including both men and women in the general population, prior foot injury significantly increased the risk of symptomatic foot OA (aOR 4.99, 95% CI 1.57–15.90) [28]. Consistent evidence across these studies highlights the long-term impact of joint injury as a risk factor for OA and emphasizes the importance of injury prevention and management.

In this study, a strong association was identified between self-reported foot/ankle injury and foot/ankle OA, and this finding aligns with the pathophysiological understanding that severe acute injury can cause destabilization of the joint, causing abnormal mechanical loading and direct articular damage, which may lead to subsequent OA changes [1, 4]. In addition to the high acute injury risk in professional footballers, repetitive use of the foot/ankle joints as well as the mechanics of movement and loading in different directions, may lead to repetitive microtrauma of joint tissues [4]. Although such microtrauma may not cause symptoms or problems at the time, the cumulative effects of this lesser injury over a professional footballer’s career may eventually result in foot/ankle OA in later life [4, 35, 36]. These findings suggest that repetitive loading, axial stress, and injuries from football activities (e.g. jumping, twisting, turning) may contribute to the development of foot/ankle OA [1, 2, 4].

Peri-articular and intra-articular injection of corticosteroids and other agents are often administered for pain relief following injury, to reduce persisting symptoms and enable players to return to play sooner [8]. However, resuming high-intensity activity before the joint has fully healed may contribute to long-term joint damage, as the injection masks the pain and permits increased activity [7]. Incomplete recovery may make the joint more vulnerable and lead to chronic instability, altered biomechanics, and cartilage damage, all of which are known pathways to OA development [1, 6]. Additionally, pain relief from injection may mask underlying joint pathology, increasing the likelihood of recurrent microtrauma and further joint damage [9]. As a result, injection may become a risk factor, independent of injury, to increase the odds of foot/ankle OA (as demonstrated in the logistic model) and add to the discrimination power (as demonstrated in the ROC analysis). In addition, the bias of confounding by indication (i.e. injection was indicated by injury) cannot be ruled out from this study [37]. However, footballers with foot/ankle OA reported a higher maximum number of injections into a single ankle during one season, and the fact that many footballers received >4 injections in a season, which is more than the currently recommended maximum number of 4 corticosteroid injections per year [38, 39], is a cause for concern. The potential harm of such a high number of corticosteroid injections into the same joint during a short-time period deserves further investigation.

### Clinical implications

Preventing foot/ankle injuries in professional footballers remains a key clinical priority, given their strong association with subsequent OA in retired UK male professional footballers. Programmes such as those focused on strengthening, proprioception, and neuromuscular training that have been shown to reduce lower limb injury risk, might be considered in this respect [5, 40].

The findings of this study raise a question concerning the frequent, and often repeated, use of corticosteroid ankle injections several decades ago when the retired footballers in this study played professionally. It is worth reviewing this treatment for acute ankle injury in current footballers to ensure that the latest guidelines for the maximum number of injections are now being followed [38, 39]. Further study is warranted to test the association between intra-articular foot/ankle injection and foot/ankle OA and to determine a safe threshold for an evidence-based clinical guideline that balances both short-term and long-term benefits for joint health.

### Limitations

This study has several limitations. First, the questionnaire survey used to assess injury and injection retrospectively has the inherent potential for recall bias. Second, as the study was focused specifically on foot/ankle OA, footballers with significant foot/ankle injuries may have been more likely to participate in the study, potentially introducing selection bias. However, no significant differences were observed between questionnaire responders and non-responders that may mitigate the risk of selection bias (Appendix 1). Third, this study is prone to misclassification bias, as foot/ankle OA was self-reported by questionnaire based on GP diagnosis or surgery at age ≥40, all of which have potential caveats concerning diagnostic accuracy for OA. A diagnosis using ROA was assessed in only 113 footballers, due to logistical reasons (a finite number of collaborating centres, and issues occurring during the COVID pandemic). However, a sensitivity analysis in those who underwent radiographic assessment showed similar sizes of ORs and discrimination powers of injury and injection as compared with those obtained from the main analysis. However, the aOR for injection was statistically insignificant (aOR 2.57, 95% CI 0.93—8.11, *P = *0.066), suggesting that the limited sample size may have reduced the statistical power to detect the difference. Further studies with larger samples are required to confirm the results for the sROA outcome.

Fourth, the study could not confirm whether the reported injections were intra-articular or peri-articular, thus further limiting interpretation of their role. We therefore conducted a subgroup analysis for the ankle joint to be more specific, and the results were similar to those of the main analysis. Furthermore, we focused on two major risk factors, injury and injection, as our primary exposures, and the rest were confounding factors. We also employed the backwards stepwise approach to select significant risk factors in the same logistic regression model to maximize power. Although the sample size calculation considered adjustment for covariates, it does not inherently eliminate multiple-testing bias. Furthermore, 16 covariates were included as confounding factors in the model, which may have led to a reduction in statistical power. Further study is needed to confirm the findings. Finally, our results were restricted to male professional footballers in the UK, and caution is needed in extrapolating the findings to female professional footballers and to other settings and populations.

## Conclusion

This study found that foot/ankle injury during their professional career was a major risk factor associated with subsequent foot/ankle OA in retired UK male professional footballers. Although injection was also found to be associated with foot/ankle OA, confounding by indication could not be ruled out. Further study is needed to test the association between intra-articular corticosteroid injection and foot/ankle OA, and whether there is a temporal or cumulative dosing effect.

The East Midlands–Leicester Central Research Ethics Committee approved the study (REC Ref: 19/EM/0354).

## Supplementary Material

keaf518_Supplementary_Data

## Data Availability

De-identified participant data from this study is only available for research purposes upon request, subject to the Data Transfer Agreement (DTA) between the University of Nottingham and the data requester according to the university data protection policy and the European General Data Protection Regulation (GDPR).
