# Supplementary material for: Injury and local injection and the risk of foot/ankle osteoarthritis: a case–control study in retired UK male professional footballers
Source: Rheumatology (Oxford). 2025 Oct 22;65(1):keaf518. doi: 10.1093/rheumatology/keaf518 (PMC12862397; doi:10.1093/rheumatology/keaf518)
Supplement: keaf518_Supplementary_Data [file keaf518_supplementary_data.docx]

# **Supplementary Table S1. Comparison between** **questionnaire responders and non-responders in retired male professional footballers.**

|  | **Questionnaire** | |  |
| --- | --- | --- | --- |
|  | **Responders** | **Non-responders** | **p-value** |
| **Footballers** | **(n=468)** | **(n=410)** |  |
| Age (years), mean (SD) | 63.70 (10.47) | 63.09 (12.27) | 0.436^a^ |
| Body mass index (kg/m2), mean (SD) | 27.22 (2.89) | 27.48 (3.13) | 0.215^a^ |
| Socioeconomic decile, median (IQR) | 8.00 (6-10) | 8.00 (6-9) | 0.187^b^ |

a = Independent sample T-test.

b = Mann-Whitney U test.

IQR = Interquartile range.

SD = standard deviation.

n = number.

# **S****upplementary Table S2. Multivariable logistic regression model for symptomatic radiographic foot/ankle OA in footballers ‎(backwards stepwise selection model).‎**

|  | Adjusted OR (95% CI) | p-value |
| --- | --- | --- |
| Foot/ankle injury | 3.33 (1.31 - 8.45) | 0.011* |
| Foot/ankle injection* | 2.57 (0.93 - 8.11) | 0.066 |

* Results derived from the last step when this variable remained in the model.

# **Supplementary Table S3. Area under the curve (AUC) for different risk factors included in the logistic regression model for symptomatic radiographic foot/ankle OA.**

| **Risk factors included in the logistic regression model** | **AUC** | **95% CI** |
| --- | --- | --- |
| Injury | 0.646 | (0.523 - 0.769) |
| Injury and injection | 0.684 | (0.566 - 0.803) |
| Full model* | 0.783 | (0.682 - 0.884) |

The full model included all risk factors collected in this study, including injury, injection, age, BMI, socioeconomic status, Charlson Comorbidity Index, nodal OA, gout, pattern 3 of the 2D:4D digit ratio, current hallux valgus, flat feet, career training dose, career duration, total matches played, position played, and training hours per week).

# **Supplementary Table S4. Multivariable logistic regression model for self-reported ankle OA in footballers ‎(backwards stepwise selection model).‎**

|  | Adjusted OR (95% CI) | p-value |
| --- | --- | --- |
| Ankle injury | 2.77 (1.28 - 5.97) | 0.009* |
| Ankle corticosteroid injection* | 4.33 (2.02 - 9.29) | <0.001* |

* Results derived from the last step when this variable remained in the model.

# **Supplementary Table S5. Area under the curve (AUC) for different risk factors included in the logistic regression model for self-reported ankle OA.**

| **Risk factors included in the logistic regression model** | **AUC** | **95% CI** |
| --- | --- | --- |
| Injury | 0.678 | (0.595 - 0.761) |
| Injury and injection | 0.751 | (0.683 - 0.820) |
| Full model* | 0.813 | (0.748 - 0.878) |

*The full model included all risk factors collected in this study, including ankle injury, injection, age, BMI, socioeconomic status, Charlson Comorbidity Index, nodal OA, gout, pattern 3 2D:4D digit ratio, current hallux valgus, flat feet, career training dose, career duration, total matches played, position played, and training hours per week.

# **Supplementary Table S6. Multivariable logistic regression model for self-reported GP-diagnosed foot/ankle OA alone in footballers ‎(backwards stepwise selection model).‎**

|  | Adjusted OR (95% CI) | p-value |
| --- | --- | --- |
| Foot/ankle injury | 3.35 (1.47 - 7.63) | 0.004* |
| Foot/ankle injection* | 2.57 (1.12 - 5.89) | 0.025* |

* Results derived from the last step when this variable remained in the model.
